# Using large administrative data for mining patients’ trajectories for risk stratification: An example from urological diseases

**DOI:** 10.1371/journal.pone.0310981

**Published:** 2024-11-13

**Authors:** Harvey Jia Wei Koh, Dragan Gašević, David Rankin, Mark Frydenberg, Stella Talic

**Affiliations:** 1 Centre for Learning Analytics, Faculty of Information Technology, Monash University, Clayton, Australia; 2 Digital Health Cooperative Research Centre, Sydney, Australia; 3 School of Public Health and Preventative Medicine, Monash University, Melbourne, Australia; 4 Cabrini Healthcare, Malvern, Australia; 5 Department of Surgery, Faculty of Medicine, Nursing and Health Sciences, Monash University, Melbourne, Australia; 6 Cabrini Institute, Cabrini Healthcare, Malvern, Australia; Tokyo Women’s Medical University, JAPAN

## Abstract

**Objective:**

To identify latent clusters among urological patients by examining hospitalisation rate trajectories and their association with risk factors and outcome quality indicators.

**Materials and methods:**

Victorian Admitted Episodes Dataset, containing information on all hospital admissions in Victoria from 2009 to 2019. The top twenty ICD-10 primary diagnosis codes in urology were used to select patients (n = 98,782) who were included in the study. Latent class trajectory modelling (LCTM) was used to cluster urological patient hospitalisation trajectories. Logistic regression was used to find baseline factors that influence cluster membership, the variables tested included comorbidities, baseline diagnosis codes, and socio-demographic factors. The analysis was further stratified into non-surgical procedures and surgical procedures.

**Results:**

Five clusters of hospitalisation trajectories were identified based on clustering hospitalisation rates over time. Higher hospitalisation clusters were strongly associated with longer length of stay, higher readmission rates and higher complication rates. Higher-risk groups were strongly associated with comorbidities such as renal disease and diabetes. For surgical procedures, urological cancers (kidney, prostate and bladder cancer) and irradiation cystitis were associated with higher-risk groups. For non-surgical procedures, calculus of the bladder, urethral stricture and bladder neck obstruction were associated with higher-risk groups. For patients with two or more admissions, liver cardiovascular disease and being diagnosed with benign prostatic hyperplasia were also associated with higher risk groups.

**Conclusion:**

A novel statistical approach to cluster hospitalisation trajectories for urological patients was used to explore potential clusters of patient risks and their associations with outcome quality indicators. This study supports the observation that baseline comorbidities and diagnosis can be predictive of higher hospitalisation rates and, therefore, poorer health outcomes. This demonstrates that it is possible to identify patients at risk of developing complications, higher length of stay and readmissions by using baseline comorbidities and diagnosis from administrative data.

## Introduction

In healthcare, quality monitoring systems play a pivotal role in ensuring the delivery of excellent care to patients. One of the essential components of quality improvement is the use of quality indicators (QIs), which are quantitative measurements that gauge the quality of care within a healthcare system [1–3]. These QIs are crucial, serving as benchmarking and feedback tools and enabling healthcare professionals to accurately assess the quality of care provided, identify variations in care, and make informed clinical decisions. The development and implementation of QIs are often performed by clinical quality registries (CQRs), which store datasets for quality monitoring and improvement [4, 5]. However, CQRs can be costly to establish and maintain, face challenges in funding and resourcing, and require substantial patient coverage to be effective [5, 6]. To overcome these obstacles and leverage the wealth of data available in healthcare systems, there is a growing interest in repurposing electronic health data to enhance the quality of care [7]. By standardising QIs commonly used in hospitals and aligning them with patient trajectories and risk factors, healthcare professionals can receive more accurate feedback, leading to better quality monitoring practices.

In the speciality of urology, significant gaps exist in QI development [8]. Recent systematic reviews have identified that few indicators are validated and piloted in practice [9]. More importantly, QIs in the speciality of urology are not standardised, making it difficult for healthcare professionals to compare the quality of care delivered [9]. Having standardised QIs will account for patient risk factors and comorbidities, enabling risk stratification/adjustment and allowing for an *apples-to-apples comparison*.

By utilising latent class trajectory modelling (LCTM), a clustering method originally used in criminology [10] and behavioural research [11], patient risk clusters can be identified based on patterns of change over time. LCTM has been effectively employed in epidemiology research for conditions like asthma [12], diabetes [13], and chronic kidney disease [14]. By testing LCTM as a tool for standardising QIs and identifying risk factors associated with poorer outcomes, this study aims to improve quality monitoring practices in urology. Current methods of risk stratifying patients in urology involve the use of the Australian Refined-Diagnosis Related Group (AR-DRG) [15] while using 3x the national average length of stay to risk adjust for practice outlier detection. This method, while provides good utility to risk adjust outcome indicators, leaves more to be desired as more complex methods can be used to obtain better risk stratification groups.

To that end, the objectives of this study are as follows: (1) Identify latent cluster memberships of urological patients with varying hospitalisation rate trajectories. (2) Examine the associations between cluster membership and sociodemographic characteristics, urology diagnosis, comorbidities, and QIs. (3) Predict latent cluster membership based on baseline diagnosis and comorbidities (first admission recorded). Through these objectives, this research aims to provide valuable insights into patient risk factors and their impact on outcomes, ultimately contributing to enhanced decision-making and quality of care in urology.

## Materials and methods

### Participants/data source/variables

**Fig 1:**
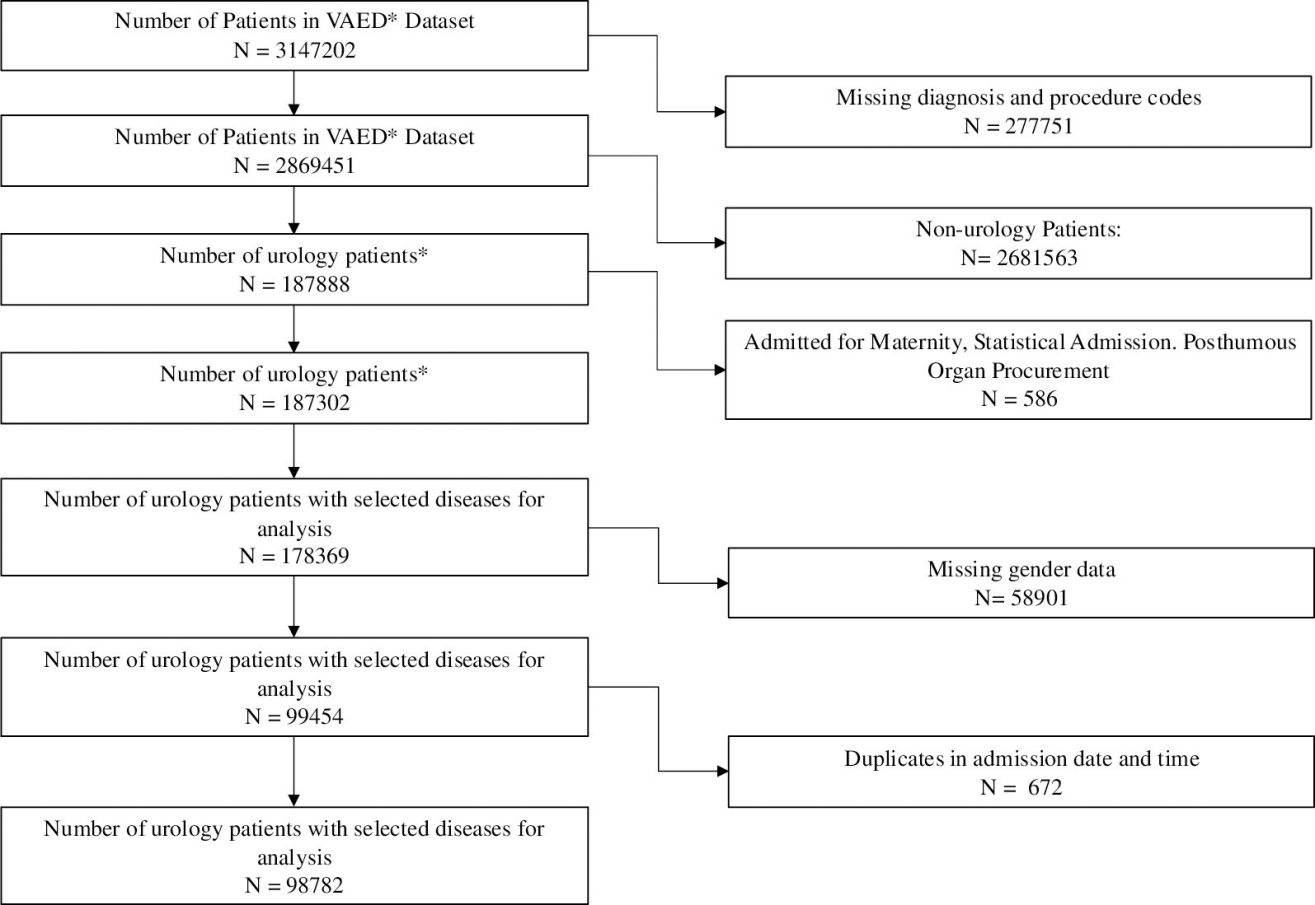
Flow chart of selected patients for analysis from Victorian Admitted Episodes Dataset.

This study utilised the Victorian Admitted Episodes Dataset (VAED), encompassing hospital admissions in Victoria from 2009 to 2019. The dataset included urological patients (n = 98,782) identified (Fig 1) based on primary urological diagnosis codes using the International Classification of Diseases 10th Edition (ICD-10) (S1 File). The top 20 (out of 135) urological ICD codes based on the prevalence of diagnosis were included in our analysis as they had the largest representation (81.2%) of admissions.

The study was approved by the ethics committee of the Alfred Hospital and Monash University under low-risk ethics, and informed consent was obtained at the time of data collection by hospital sites for secondary use of research. The study reference number is 138–20. The study was performed in accordance with ethical guidelines and the Declaration of Helsinki. In accordance with legal data protection requirements, only de-identified data had been used. The data was accessed on the 23^rd^ of August, 2023.

Several outcome measures were considered for identifying latent cluster membership: (1) cumulative hospitalisation rate, (2) cumulative complication rate and (3) readmission rate. (4) cumulative length of stay. Cumulative hospitalisation rate was chosen as the latent cluster variable, given the previous study that suggests its direct relationship with multiple outcome variables [14]. Candidate outcome variables such as complication rates and readmission rates were excluded due to sparsity leading to unstable clusters. Cumulative length of stay was excluded due to the lack of heterogeneity of clusters, leading to poor stratification of risk groups. The hospitalisation rate was calculated based on the length of stays exceeding one day. The first three years’ rates were chosen as the analysis endpoint because patient hospitalisation history exceeding three years was limited.

Demographic variables such as age, insurance status, English-speaking, culturally and linguistically diverse (CALD) and Indigenous groups, as well as the patient’s rurality (urban, regional, rural), were included in the analysis.

Comorbidities were identified using ICD-10 diagnosis codes and identified based on the Charlson method [16]. Cardiovascular diseases, diabetes, liver disease, and renal disease were considered comorbidities during analysis. Cancer was excluded during the model-building process to prevent confounding with the primary diagnosis of cancer.

Quality indicators and hospital-acquired complications from the VAED dataset were analysed. These included length of stay per admission, 7-day unplanned readmissions, 28-day unplanned readmissions, and the number of hospital-acquired complications (HACs) [17]. HACs were defined using specific rulesets from the Australian Commission on Safety and Quality in Health Care (ACSQHC) [18].

### Statistical analysis

#### Latent class trajectory modelling

All statistical analyses based on latent class trajectory modelling (LCTM) were performed using the R package *lcmm*. LCTM seeks to simplify heterogeneous populations into homogenous clusters. The data was presented as a time series of cumulative hospitalisations for each urology patient and fitted with a latent class linear mixed model (*hlme* function in *lcmm* package). Using a previously developed framework for latent class trajectory modelling [19, 20], the first step was to determine the initial working model based on the residual profile, Bayes information criterion (BIC), and residual standard error. Four models with increasing amounts of polynomials (linear, quadratic, cubic and quartic) were tested. It was found that while a quartic polynomial had a lower BIC, the decrease in the standard error of residuals was marginal (S2 File). Hence, cubic representation was chosen as the base model.

The optimal number of classes (groups) was tested, with testing of one to eight total classes. To ensure that the model chosen was reflective of the data, several model adequacy tests were performed. While the lowest BIC has largely been used as a tool for model selection [10], in some cases, overfitting may cause BIC to decrease further. Hence, other model adequacy methods were also considered [19]. In this study, apart from BIC, five main criteria were considered as part of the model adequacy test: (1) A maximum posterior probability of assignments (APPA) above 70% for all classes, indicating that each member has been assigned to the right class. (2) Odds of correct classification above 5.0 for each class for indicating that there is strong discrimination between classes. (3) A mismatch of close to zero for each class that would indicate a precise model. (4) A relative entropy of close to 1, which would indicate well-separated classes. (5) An entropy of close to 0, indicating high certainty in classifying patients in separate groups. These five criteria are used to assess and balance model fit (BIC) against meaningful trajectories, model parsimony and model adequacy [19].

Based on the fit criteria and model adequacy tests, five different hospitalisation groups were identified, which were assigned based on the posterior probabilities for each group for each patient identified (S2 File) [19].

For sensitivity analysis, a sub-analysis of patients with two or more admissions to enable a time series assessment and understand if any differences exist between the sub-group of patients with two or more admissions when compared to the patient sample (S3 File). Sensitivity analysis was included to ensure that the first group with the lowest hospitalisations are not falsely grouped due to having one admission throughout the study period and are patients who repeatedly have very low cumulative hospitalisation rates.

## Identifying significant covariates

Next, similar to other studies [14, 21], the descriptive statistics for each patient group were summarised based on mean ± standard deviation (SD) or median and interquartile range (IQR) for continuous variables and as percentages for frequency distributions for categorical variables. For skewed distributions, we performed a natural logarithmic transformation. Chi-squared tests were used to evaluate the frequency distribution of categorical variables, while continuous variables were evaluated using analysis of variance and the Kruskal-Wallis test.

## Model inference and risk stratification using logistic regression

Finally, to understand how variables can affect membership of groups. All covariates were tested with a multinomial logistic regression to assess the association between group membership and sociodemographic characteristics, urology diagnosis, and comorbidities during the first admission to the hospital. Surgical procedures were also identified based on ACHI code blocks, and interactions with urology diagnosis were considered.

For more comprehensive risk stratification, admissions information such as emergency/elective admissions, as well as the presence of HACs in the first admission, and interactions between diagnosis and the presence of surgical interventions were also included. The presence of surgical intervention was determined using the Australian Classification of Healthcare Interventions (ACHI), with intervention codes belonging to radio-oncology, non-invasive interventions and imaging services being classified as non-surgical procedures [22].

Data cleaning, feature generation, and statistical analysis were run in Python, except for the LCTM model, which was run on the R package *lcmm*.

## Results

### Cumulative all-cause hospitalisation trajectories

**Fig 2:**
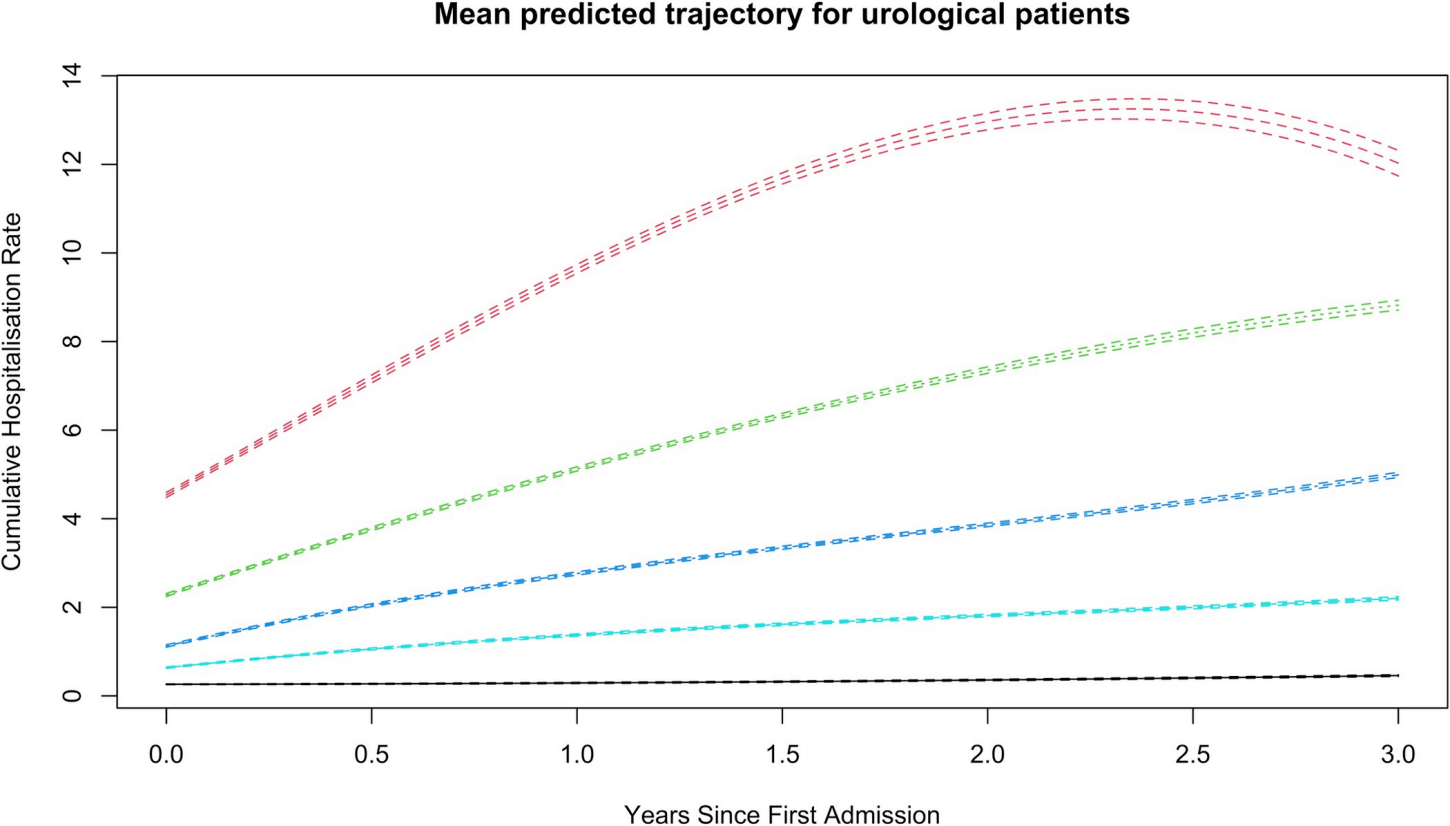
Fitted trajectories for each class, indicating the trajectory of total hospitalisations that a urology patient may exhibit within three years of first hospitalisation, clustered into five groups of no (class I), low (class II), medium (class III), medium-high hospitalisations (class V). Note: The large confidence interval for class V post-two years is due to the low sample size.

The outcome of our LCTM analysis of hospitalisation trajectories is presented in Fig 2. A five-group model with cubic splines for all groups was chosen. The identified groups were labelled based on their cumulative hospitalisation rates: class I (n = 86097, 87.16%), class II (n = 4937, 5.0%), class III (n = 5559,5.63%), class IV (n = 1688,1.71%), class V (n = 501,0.51%). The model showed that APPA was mostly above 70% for each group, had high OCC (above 7) for all classes, had a very low level of mismatch and had a better fit based on BIC.

### Patient characteristics and covariate relationship with group membership

There was a total of 98,782 patients included in the analysis for this study (Table 1). In comparison to patients in class I (low hospitalisation), patients in class II (medium-low hospitalisation), class III (medium hospitalisation), class IV (medium-high hospitalisation) and class V (high hospitalisation) were likely to have a higher Charlson score (mean ± SD, class I: 3.0 ± 5.1, class II: 5.3 ± 5.8, class III:7.5 ± 7.6, class IV: 10.3 ± 7.2, class V: 12.1 ± 7.1), older (mean ± SD, class I: 64.2 ± 16.5, class II: 70.3 ± 13.1, class III: 73.4 ± 13.3, class IV: 73.3 ± 13.2, class V: 70.9 ± 15.3), more likely to be male, English speaking, non-culturally and linguistically diverse, have no hospital insurance, have a larger prevalence of diabetes, cardiovascular disease, liver disease, renal disease or have two or more comorbidities. Patients who are in higher risk groups also tend to be admitted as emergency patients rather than elective, have had surgical procedures and likely to have HACs and come from rural areas.

**Table 1:** Patient characteristics of different hospitalisation groups.

Patient Characteristics	Class I, Low hospitalisation (n = 86097, 87.16%)	Class II, Medium-low hospitalisation (n = 4937,5.0%)	Class III, Medium hospitalisation (n = 5559,5.63%)	Class IV, Medium-high hospitalisation (n = 1688,1.71%)	Class V, High hospitalisation (n = 501,0.51%)	P-value
**Hospitalisation per year, mean ± SD**	2.9±6.6	3.9±5.0	7.3±7.2	8.2±6.4	9.1±5.3	**<0.05**
**Charlson Score (Age Unadjusted), mean ± SD**	3.0±5.1	5.3±5.8	7.5±7.6	10.3±7.2	12.1±7.1	**<0.05**
**Demographics**						
Age, mean ± SD	64.2±16.5	70.3±13.1	73.4±13.3	73.3±13.2	70.9±15.3	**<0.05**
Male	67822 (78.8)	4372 (88.6)	14775 (85.9)	1438 (85.2)	420 (83.8)	**< 0.001**
**English Speaking (%)**						**<0.001**
Yes	76712 (89.1)	4326 (87.6)	4921 (88.5)	1478 (87.6)	448 (89.4)	
No	9385 (10.9)	611 (12.4)	638 (11.5)	210 (12.4)	53 (10.6)	
**Rurality**						
Urban	59592 (69.2)	3336 (67.6)	3769 (67.8)	1168 (69.2)	336 (67.1)	**<0.001**
Regional	8630 (10.0)	482 (9.8)	535 (9.6)	144 (8.5)	40 (8.0)	
Rural	17875 (20.8)	1119 (22.7)	1255 (22.6)	376 (22.3)	125 (25.0)	
CALD (%) ^						**<0.05**
Yes	10230 (11.9)	653 (13.2)	680 (12.2)	223 (13.2)	57 (11.4)	
No	75867 (88.1)	4284 (86.8)	4879 (87.8)	1465 (86.8)	444 (88.6)	
**Indigenous (%)**						**0.401**
Yes	868 (1.0)	44 (0.9)	44 (0.8)	13 (0.8)	4 (0.8)	
No	85229 (99.0)	4893 (99.1)	5515 (99.2)	1675 (99.2)	497 (99.2)	
**Hospital Insurance Status (%)**						**<0.001**
Yes	37528 (43.6)	2340 (47.4)	2366 (42.6)	663 (39.3)	203 (40.5)	
No	44451 (51.6)	2418 (49.0)	2943 (52.9)	963 (57.0)	280 (55.9)	
Unknown	4118 (4.8)	179 (3.6)	250 (4.5)	62 (3.7)	18 (3.6)	
**Comorbidities at baseline (%)**						**<0.001**
Diabetes	12954 (15.0)	914 (18.5)	1085 (19.5)	296 (17.5)	76 (15.2)	
Renal	810 (0.9)	99 (2.0)	228 (4.1)	92 (5.5)	32 (6.4)	
Two or more comorbidities	1991 (2.3)	172 (3.5)	387 (7.0)	108 (6.4)	31 (6.2)	
Cardiovascular disease	1081 (1.3)	78 (1.6)	191 (3.4)	51 (3.0)	18 (3.6)	
Liver disease	97 (0.1)	5 (0.1)	13 (0.2)	6 (0.4)	1 (0.2)	
**Admission status at baseline (%)**						**<0.001**
Elective	73492 (85.4%)	3970 (80.4%)	3196 (57.5%)	949 (56.2%)	252 (50.3%)	
Emergency	12605 (14.6%)	967 (19.6%)	2363 (42.5%)	739 (43.8%)	249 (49.7%)	
**Complications at baseline (%)**						**<0.001**
No	82229 (95.5%)	4530 (91.8%)	4778 (86.0%)	1419 (84.1%)	423 (84.4%)	
Yes	3868 (4.5%)	407 (8.2%)	781 (14.0%)	269 (15.9%)	78 (15.6%)	
**Surgical procedure at baseline (%)**						**<0.001**
No	73717 (85.6%)	4474 (90.6%)	5297 (95.3%)	1601 (94.8%)	475 (94.8%)	
Yes	12380 (14.4%)	463 (9.4%)	262 (4.7%)	87 (5.2%)	26 (5.2%)	
**Diagnosis at Baseline (%)**						**< 0.05**
Prostate cancer	10848 (12.6)	1170 (23.7)	1194 (21.5)	572 (33.9)	171 (34.1)	
Kidney cancer	2366 (2.7)	253 (5.1)	516 (9.3)	249 (14.8)	111 (22.2)	
Bladder cancer	2792 (3.2)	427 (8.6)	505 (9.1)	207 (12.3)	72 (14.4)	
Two or more urological conditions	7985 (9.3)	647 (13.1)	615 (11.1)	144 (8.5)	35 (7.0)	
Benign prostatic hyperplasia	15576 (18.1)	902 (18.3)	1161 (20.9)	179 (10.6)	31 (6.2)	
Testicular cancer	548 (0.6)	13 (0.3)	35 (0.6)	27 (1.6)	31 (6.2)	
Calculus of kidney	8274 (9.6)	326 (6.6)	321 (5.8)	98 (5.8)	14 (2.8)	
Other specified disorders of bladder	8476 (9.8)	220 (4.5)	220 (4.0)	52 (3.1)	10 (2.0)	
Calculus of ureter	7892 (9.2)	191 (3.9)	222 (4.0)	37 (2.2)	6 (1.2)	
Irradiation cystitis	343 (0.4)	27 (0.5)	35 (0.6)	13 (0.8)	5 (1.0)	
Calculus of kidney with calculus of ureter	1569 (1.8)	80 (1.6)	87 (1.6)	10 (0.6)	5 (1.0)	
Other specified urinary incontinence	3311 (3.8)	102 (2.1)	139 (2.5)	15 (0.9)	3 (0.6)	
Cystitis, unspecified	2062 (2.4)	50 (1.0)	72 (1.3)	14 (0.8)	2 (0.4)	
Hydrocele, unspecified	1815 (2.1)	35 (0.7)	41 (0.7)	7 (0.4)	1 (0.2)	
Disorders of prepuce	2998 (3.5)	30 (0.6)	50 (0.9)	9 (0.5)	1 (0.2)	
Calculus in bladder	1064 (1.2)	61 (1.2)	68 (1.2)	17 (1.0)	1 (0.2)	
Other specified disorders of male genital organs	2138 (2.5)	49 (1.0)	56 (1.0)	8 (0.5)	1 (0.2)	
Urethral stricture, unspecified	4528 (5.3)	274 (5.5)	164 (3.0)	23 (1.4)	1 (0.2)	
Other specified disorders of penis	622 (0.7)	20 (0.4)	24 (0.4)	2 (0.1)	0 (0.0)	
Bladder-neck obstruction	890 (1.0)	60 (1.2)	34 (0.6)	5 (0.3)	0 (0.0)	
**Quality Indicators**						
Length of Stay per admission, days, mean ± SD	2.9±6.6	3.9±5.0	7.3±7.2	8.2±6.4	9.1±5.3	**<0.05**
Complications per year, mean ± SD	0.5±1.6	1.1±1.9	2.8±3.8	5.2±5.2	8.1±7.4	**<0.05**
7-day readmission per year, mean ± SD	0.0±0.1	0.0±0.1	0.1±0.2	0.1±0.4	0.3±0.6	**<0.05**
28-day readmission per year, mean ± SD	0.0±0.1	0.0±0.2	0.2±0.4	0.5±0.7	1.1±1.1	**<0.05**

^ CALD: culturally and linguistically diverse.

Patients in higher risk groups (class II, III, IV, V) were likely to have longer lengths of stays (mean ± SD, class I: 2.9 ± 6.6, class II: 3.9 ± 5.0, class III: 7.3 ± 7.2, class IV: 8.2 ± 6.4, class V: 9.1 ± 5.3) and have more complications per year (mean ± SD, class I: 0.5 ± 1.6, class II: 1.1 ± 1.9, class III: 2.8 ± 3.8, class IV: 5.2 ± 5.2, class V: 8.1±7.4). Furthermore, compared to class I, which had no readmissions, 7-day and 28-day readmissions were significantly higher for higher-risk classes.

### Predictors of group membership using multivariate multinomial logistic regression

#### Baseline demographics, admission factors and comorbidities

**Fig 3:**
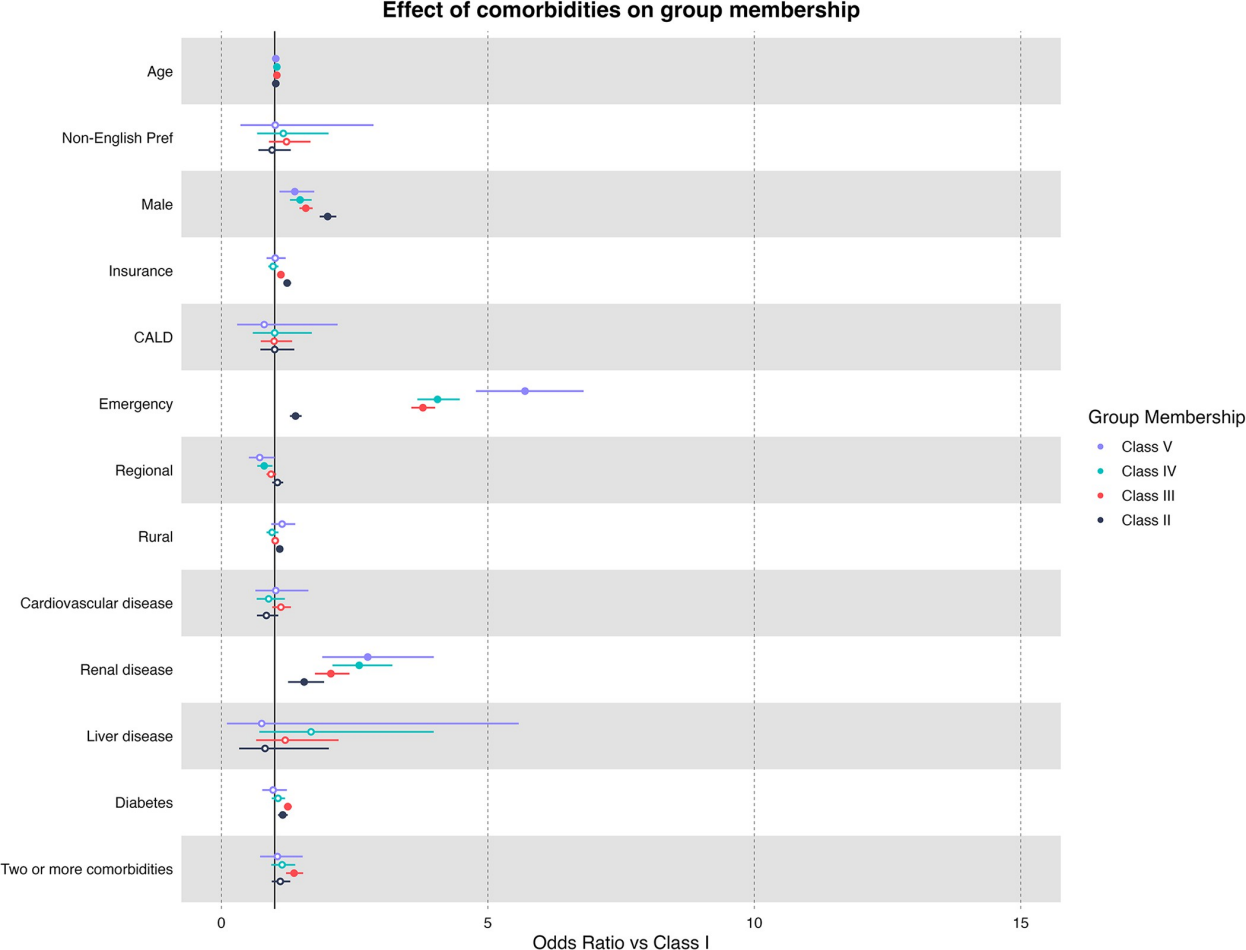
Multiple multinomial logistic regression results and odds ratio for comorbidities diagnosed for first admission. * Bold indicates significant associations (p <0.05), hollow dots indicate non-significance (p > 0.05). Each comorbidity is compared to other patients in the pool, i.e., Renal disease patients have a higher risk of being in class III, IV, and V when compared to other urological patients.

In our multivariate multinomial logistic regression adjusted for demographic factors (age, gender, insurance status, non-English preference, CALD and rurality), patients who were admitted as an emergency admission had significantly higher odds of being classified as class II or higher (Class II: OR = 1.35 [95% CI: 1.25–1.46], Class III: OR = 3.63 [95% CI: 3.43–3.85], Class IV: OR = 3.82 [95% CI: 3.46–4.21], Class V: OR = 5.47 [95% CI: 4.59–6.53]) (Fig 3). Furthermore, patients who were afflicted with HACs were found to be higher odds of classified as class II or higher (Class II: OR = 1.35 [95% CI: 1.25–1.46], Class III: OR = 3.63 [95% CI: 3.43–3.85], Class IV: OR = 3.82 [95% CI: 3.46–4.21], Class V: OR = 5.47 [95% CI: 4.59–6.53]).

Comorbidities were also found to be associated with higher-risk groups. It was found that relative to the baseline class (class I), patients of class III, IV, V were significantly likely of having renal disease (Class II: OR = 1.43 [95% CI: 1.16–1.78], Class III: OR = 1.84 [95% CI: 1.57–2.15], Class IV: OR = 2.25 [95% CI: 1.78–2.84], Class V: OR = 2.39 [95% CI: 1.61–3.53]). Patients with diabetes were associated with class II, class II and class IV (Class II: OR = 1.14 [95% CI: 1.05–1.23], Class III: OR = 1.23 [95% CI: 1.16–1.31], Class IV: OR = 1.05 [95% CI: 0.93–1.18], Class V: OR = 0.95 [95% CI: 0.75–1.2]). While non-significant, patients with liver disease were found to be associated with class III and class IV risk groups (Class II: OR = 0.76 [95% CI: 0.31–1.88], Class III: OR = 1.09 [95% CI: 0.6–2.01], Class IV: OR = 1.48 [95% CI: 0.62–3.5], Class V: OR = 0.65 [95% CI: 0.09–4.9]).

## Non-surgical interventions at baseline

**Fig 4:**
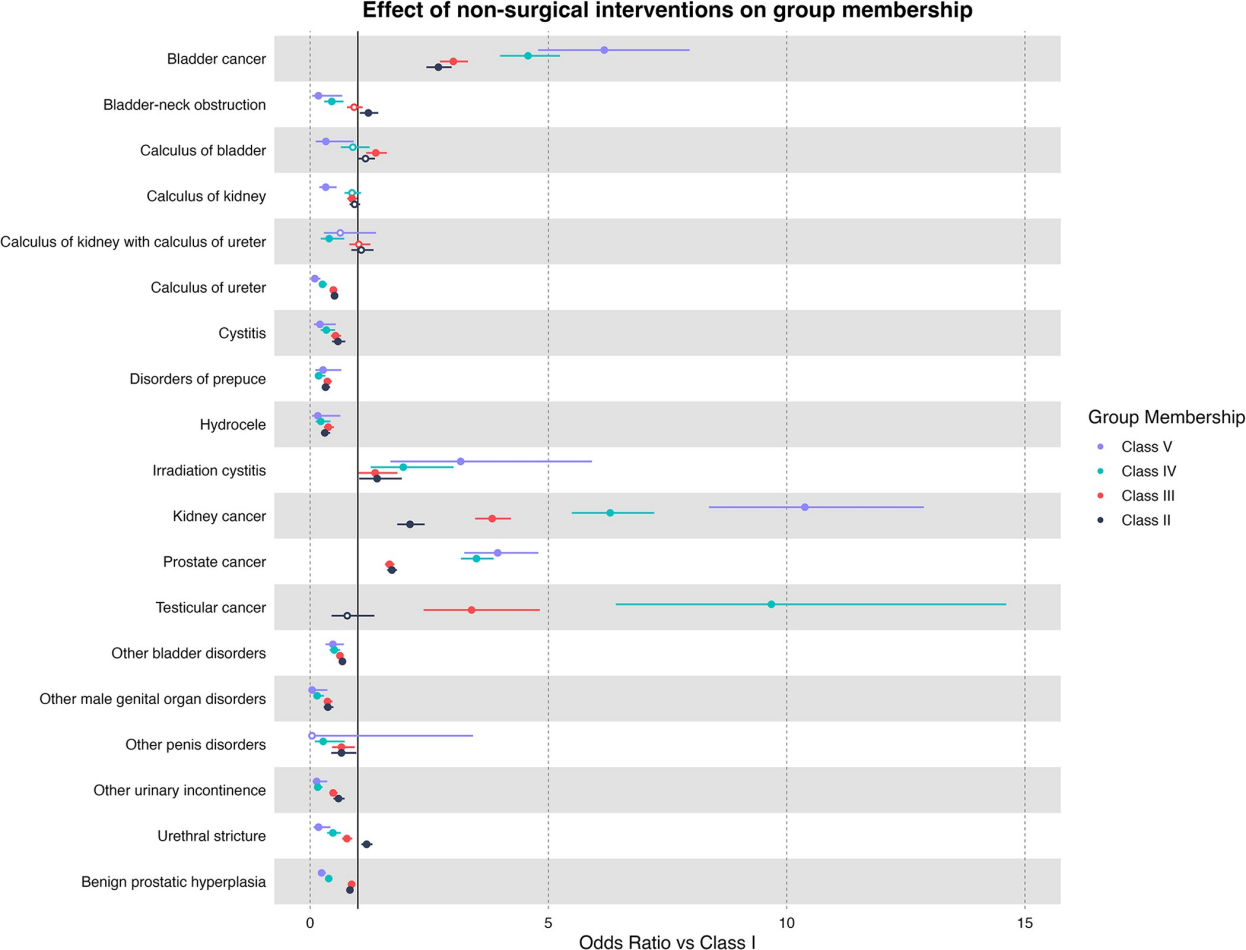
Multiple multinomial logistic regression results and odds ratio for non-surgical urological diseases diagnosed for first admission. * Bold indicates significant associations (p <0.05), hollow dots indicate non-significance (p > 0.05). Each disease is compared to other patients in the pool, i.e., bladder cancer patients have a higher risk of being in class II, III, IV, and V when compared to other urological patients.

Overall, patients being diagnosed with bladder cancer, kidney cancer, prostate cancer and testicular cancer at baseline had the highest risk of being classified higher than class I when compared to other urological diagnoses (Fig 4). In particular, testicular cancer has the highest odds of being classified as class III, class IV, or class V (Class II: OR = 0.78 [95% CI: 0.45–1.35], Class III: OR = 3.39 [95% CI: 2.38–4.82], Class IV: OR = 9.68 [95% CI: 6.41–14.61], Class V: OR = 32.79 [95% CI: 21.3–50.46]). Followed by kidney cancer (Class II: OR = 2.1 [95% CI: 1.83–2.4], Class III: OR = 3.82 [95% CI: 3.46–4.21], Class IV: OR = 6.3 [95% CI: 5.49–7.22], Class V: OR = 10.38 [95% CI: 8.37–12.88]), bladder cancer (Class II: OR = 2.69 [95% CI: 2.44–2.97], Class III: OR = 3.0 [95% CI: 2.72–3.31], Class IV: OR = 4.57 [95% CI: 3.99–5.24], Class V: OR = 6.17 [95% CI: 4.78–7.96]) and prostate cancer (Class II: OR = 1.72 [95% CI: 1.62–1.82], Class III: OR = 1.67 [95% CI: 1.57–1.77], Class IV: OR = 3.49 [95% CI: 3.16–3.85], Class V: OR = 3.94 [95% CI: 3.23–4.79]). Irradiation cystitis also has higher odds of being classified as class II or higher (Class II: OR = 1.4 [95% CI: 1.03–1.92], Class III: OR = 1.36 [95% CI: 1.02–1.83], Class IV: OR = 1.95 [95% CI: 1.27–3.01], Class V: OR = 3.16 [95% CI: 1.69–5.91]).

When compared to other diagnosis, calculus of bladder had higher odds of being classified as class III (Class II: OR = 1.16 [95% CI: 0.99–1.36], Class III: OR = 1.38 [95% CI: 1.18–1.61], Class IV: OR = 0.9 [95% CI: 0.64–1.25], Class V: OR = 0.33 [95% CI: 0.12–0.91]), while urethral stricture class II (Class II: OR = 1.19 [95% CI: 1.07–1.31], Class III: OR = 0.77 [95% CI: 0.67–0.88], Class IV: OR = 0.48 [95% CI: 0.36–0.64], Class V: OR = 0.18 [95% CI: 0.07–0.42]) and bladder neck obstruction (Class II: OR = 1.22 [95% CI: 1.04–1.43], Class III: OR = 0.92 [95% CI: 0.77–1.1], Class IV: OR = 0.45 [95% CI: 0.29–0.7], Class V: OR = 0.17 [95% CI: 0.04–0.67]) has higher odds of being classified as class II. All other urological diagnosis has lower odds of being classified as class II or higher.

## Surgical interventions at baseline

**Fig 5:**
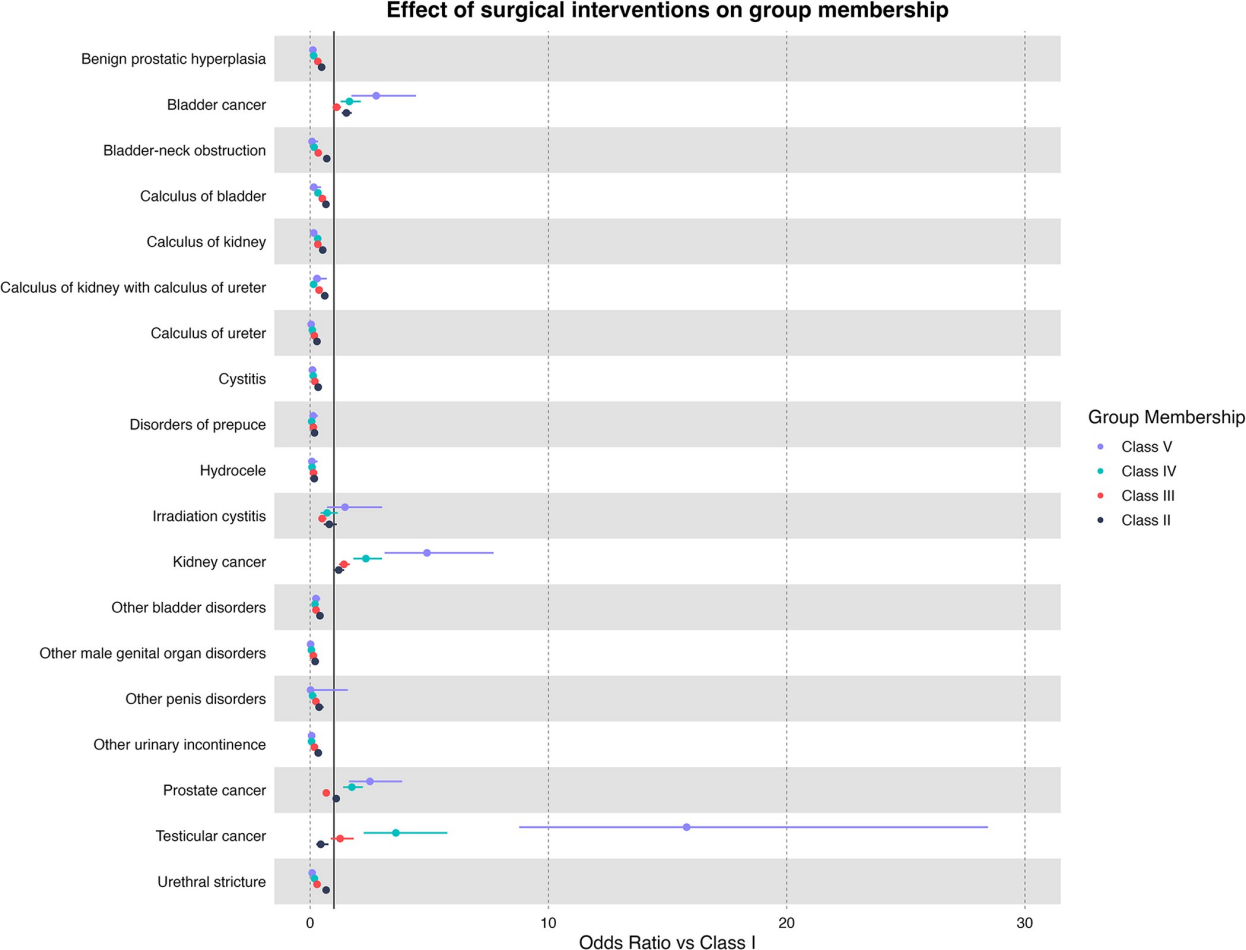
Multiple multinomial logistic regression results and odds ratio for urological diseases diagnosed for first admission with surgical interventions. *Bold indicates significant associations (p <0.05), hollow dots indicate non-significance (p > 0.05). Each disease is compared to other patients in the pool, i.e., bladder cancer patients have a higher risk of being in class II, III, IV, and V when compared to other urological patients.

The odds ratio of each diagnosis at baseline compared to the rest of the urological patients is presented in Fig 5. Overall, testicular cancer patients (Class II: OR = 0.44 [95% CI: 0.26–0.77], Class III: OR = 1.26 [95% CI: 0.87–1.83], Class IV: OR = 3.6 [95% CI: 2.25–5.76], Class V: OR = 15.8 [95% CI: 8.78–28.45]) were the most likely to be classified as class II or higher when compared to the other diseases. Followed by kidney (Class II: OR = 1.2 [95% CI: 1.0–1.43], Class III: OR = 1.42 [95% CI: 1.21–1.66], Class IV: OR = 2.34 [95% CI: 1.81–3.02], Class V: OR = 4.9 [95% CI: 3.12–7.7]), bladder (Class II: OR = 1.52 [95% CI: 1.33–1.75], Class III: OR = 1.11 [95% CI: 0.94–1.29], Class IV: OR = 1.65 [95% CI: 1.28–2.13], Class V: OR = 2.77 [95% CI: 1.73–4.44]) and prostate cancer (Class II: OR = 1.09 [95% CI: 0.97–1.23], Class III: OR = 0.68 [95% CI: 0.59–0.78], Class IV: OR = 1.75 [95% CI: 1.38–2.21], Class V: OR = 2.51 [95% CI: 1.63–3.86]).

With surgical intervention, irradiation cystitis has higher odds of being classified as class V (Class II: OR = 0.8 [95% CI: 0.58–1.12], Class III: OR = 0.5 [95% CI: 0.37–0.69], Class IV: OR = 0.71 [95% CI: 0.44–1.16], Class V: OR = 1.46 [95% CI: 0.71–3.02]). Other diagnoses had relatively low odds of being classified as class II or higher.

For full results on baseline, comorbidities and diagnosis, please refer to S4 File.

## Patients with two or more admissions

A sensitivity analysis of patients with two or more admissions revealed no substantial difference in the trajectories. However, with comorbidities, when compared to baseline low hospitalisation groups (class I), patients with two or more admissions also show that liver disease (Class II: OR = 19.11 [95% CI: 4.39–83.1], Class III: OR = 13.07 [95% CI: 2.89–59.1], Class IV: OR = 34.12 [95% CI: 7.11–163.69], Class V: OR = 11.36 [95% CI: 0.94–136.89]), cardiovascular diseases (Class II: OR = 3.25 [95% CI: 2.47–4.28], Class III: OR = 2.72 [95% CI: 2.03–3.65], Class IV: OR = 2.36 [95% CI: 1.63–3.43], Class V: OR = 3.03 [95% CI: 1.79–5.15]) and two or more comorbidities (Class II: OR = 3.29 [95% CI: 2.76–3.92], Class III: OR = 2.61 [95% CI: 2.19–3.12], Class IV: OR = 2.41 [95% CI: 1.87–3.11], Class V: OR = 2.53 [95% CI: 1.71–3.75]) were significantly associated with higher risk groups.

In the sensitivity analysis, patients who underwent non-surgical interventions, patients with repeated hospitalisations and those diagnosed with benign prostatic hyperplasia (BPH) were also likely to be classified as class II or class III when compared to other diagnosis groups (Class II: OR = 1.55 [95% CI: 1.44–1.68], Class III: OR = 1.72 [95% CI: 1.59–1.86], Class IV: OR = 0.73 [95% CI: 0.62–0.85], Class V: OR = 0.44 [95% CI: 0.32–0.61]). Similar to our main findings, patients with kidney, prostate, bladder and testicular cancer, as well as irradiation cystitis, were likely to be classified as class II or higher.

Similar to the main findings, patients with two or more admissions and who had undergone surgical procedures for testicular cancer, kidney cancer and bladder cancer also show higher odds of being classified as class II or higher. For the full sensitivity analysis, please refer to S5 File.

## Discussion

To our knowledge, this is the first attempt to cluster hospitalisation trajectories of urological patients by applying LCTM to a large administrative data set. The main aim of this study was to identify patient risk factors based on hospitalisation clusters and predict cluster membership predicated on baseline diagnosis and comorbidities. Our findings indicate that (a) cluster membership was predictive of worse or worsening outcomes (longer length of stay, higher unplanned re-admissions, and higher hospital-acquired complications); (b) increased hospitalisation was strongly associated with comorbidities such as liver disease, cardiovascular disease, renal disease, and diabetes; (c) patient diagnosis at baseline significantly affected their attributed group membership.

Our finding that cluster membership was predictive of worse or worsening outcomes is significant, as longer lengths of stay and unplanned readmissions have been associated with higher mortality rates in previous work [23]. Furthermore, we found that patient comorbidities are also strongly correlated with increased hospitalisation. Such findings imply that if accurate predictions of risk group membership can be obtained based on baseline information (comorbidities and diagnosis during the first visit), the associated patient risks and expected outcomes can be timely provided to healthcare professionals for feedback and early monitoring purposes, facilitating improvement of clinical outcomes and practice. It is noted that some comorbidities, such as liver disease and renal diseases, have large confidence intervals, especially in higher-risk groups, this could potentially be explained by the smaller sample sizes in the higher-risk groups, with only 0.51% of total patients being classified as high hospitalisation risk profiles. Nevertheless, the significant high odds ratio indicates that comorbidities such as liver disease, renal disease and cardiovascular disease play a significant role in increased hospitalisations over the study period [24, 25].

A recent systematic review of urological QIs showed that QIs are not standardised and rarely risk-adjusted/stratified; hence, they cannot be used for benchmarking [9]. Furthermore, current methods of risk stratifying patients in Australia, especially with diseases/patients with a lack of resources for quality improvement activities conducted by CQRs, can be sparse, particularly in non-oncology-related urology [8, 9]. In lieu of complex risk-stratifying methods, some governmental bodies and studies have utilised the AR-DRG code as a way of risk-stratifying patients [15, 26]. However, while risk stratifying using the AR-DRG code may be useful for administrative purposes and risk adjustment, the inability of healthcare professionals to further understand how the final risk groups were derived using AR-DRG means that it may be insufficient to provide feedback solely based on AR-DRG as a risk stratification tool. By using LCTM to risk stratify patients based on cumulative hospitalisation rate, which is shown to be correlated with other potential poor outcomes, risk stratification could potentially be much more accurate, as well as convey critical information regarding how each patient’s risk factors are associated with different risk groups. More comprehensive risk stratification would ensure an “*apples to apples”* comparison and prevent healthcare professionals from being unfairly penalised for treating higher-risk patients. Furthermore, in comparison to current methods, accurate information on the quality of care delivered can be communicated to healthcare professionals.

It was also found that patients with a diagnosis of kidney cancer, testicular cancer, bladder cancer, and irradiation cystitis were more likely to be placed in a higher hospitalisation group, even after adjusting for comorbidities, especially in patients who undergo surgery. While bladder cancer and prostate cancer have well-developed QIs [9], testicular cancer lags slightly behind in development. Potentially, this means that quality improvement teams could use the model developed in this study for risk stratification of outcome QIs in testicular cancer to ensure parity when comparing heterogeneous patients with varying risk factors.

Although kidney cancer is one of the most commonly diagnosed cancers worldwide, [27] with high complication rates and length of stay [28], kidney cancer QIs are still underdeveloped [29]. In 2017, it was noted that the development of validated QIs in kidney cancer has lagged behind other tumour sites such as colorectal cancer and breast cancer [29]. In recent years, a proliferation of QIs developed by governments and CQRs has resulted in more QIs being used for improving care for kidney cancer [30, 31]. Given that the development of QIs in kidney cancer is fairly new and lacks construct validity [29], further studies could also explore the use of LCTM as a standardisation tool.

Irradiation cystitis was also found to be a high-risk diagnosis. In particular, haemorrhagic cystitis after radiation therapy for prostate cancer has been found in the literature to be associated with high morbidity and complication rates [32]. Currently, there remain very few QIs in uro-oncology that explicitly mention irradiation cystitis as a complication to be monitored [9]. Given that our findings suggest that there will be increased hospitalisation for patients with irradiation cystitis, further monitoring of patients with irradiation cystitis may be warranted.

Our sensitivity analysis also elucidated some interesting findings on patients diagnosed with BPH, one of the most common urological diseases [33]. Patients having BPH with more than one visit have higher odds of being classified as a higher risk patient. Given the lack of focus on QIs in BPH [9], improving the quality of care monitoring system surrounding BPH, particularly for patients who have two or more visits, may be warranted.

Beyond its clinical findings, this study also presents LCTM as a potential tool to be used in wider quality improvement, allowing healthcare professionals to identify potential trends of poorer outcomes prior to further progression. This study shows that patient baseline characteristics can be used to predict the potential trajectory of a patient. Patients who fit the medium (class III), medium-high (class IV) and high hospitalisation (class V) profiles would potentially benefit from early monitoring for complications and worsening health outcomes. Care should especially be taken to ensure that higher-risk patients do not suffer from HACs, as this study has shown that patients who suffer from HACs tend to have poorer outcomes and are often classified as higher hospitalisation groups. By understanding which risk group each patient belongs to, not only can healthcare professionals be more informed on the potential outcome of the patient in the long term but can also take more proactive measures in ensuring that all patients, particularly ones in higher risk groups, do not suffer from avoidable harm.

This study also shows the potential use of LCTM as a clustering tool to identify and risk predict patients of differing outcome trajectories by attributing baseline patient characteristics, comorbidities, and diagnosis to the potential outcome trajectory of the patient. On a larger scale, methods suggested here could potentially be used to standardise QIs for different diseases for benchmarking, with a focus on diseases (due to low prevalence or low burden of disease) that may not have the resources to maintain a complete set of QIs developed and managed by CQRs. Future studies should explore the use of LCTM clusters as a potential tool for standardising QIs and assessing risks in patients, which not only considers baseline characteristics and current risk factors but also the predicted trajectory of the patient.

The potential limitations of our study include, Firstly, the use of administrative data for determining diagnosis and comorbidities, which has been shown at times to be somewhat inaccurate due to coding errors and missing data due to incompleteness of data entry [34]. Care is taken to ensure that all data has been cleaned and standardised before analysis. Secondly, patients’ data recorded were assumed to not have any other hospitalisations outside the state of Victoria, Australia. This may result in loss of follow-up in patients who have moved interstate or overseas. Thirdly, the loss of data due to missing gender data may result in a loss of representativeness of the data. However, it is unlikely given the large sample of urological patients extracted from this dataset, as well as testing to ensure that the original distribution of the data is not significantly different from the final data. Finally, this risk stratification method has not been validated and requires future validation to ensure generalisability.

## Conclusion

In conclusion, this study uses a novel statistical approach to cluster longitudinal hospitalisation data for urology patients to explore potential clusters of patient risks as measured by outcome measures. This study supports that baseline comorbidities and diagnosis can be predictive of higher hospitalisation rates and, therefore, poorer outcomes in the future. This study also demonstrates that it is possible to identify patients at risk of developing complications, higher length of stay, and readmissions in the future by using baseline comorbidities and diagnosis. This would enable early preventative measures to be taken when dealing with patients who belong to a high-risk group. Furthermore, by showing that outcomes of QIs are strongly associated with group membership, it is hoped that future studies could explore the potential use of group membership as a method for risk-adjusting patient outcomes to ensure accurate and valid feedback to medical practitioners to improve clinical practice and quality of care across multiple specialities and diseases.

## Supporting information

S1 File ICD codes included as urological diseases. (PDF)

S2 File Diagnostic of latent class trajectory model and model selection process. (PDF)

S3 File Diagnostic of latent class trajectory model and model selection process for patients with two or more admissions. (Sub-analysis).
(PDF)

S4 File Full results of baseline, comorbidity and diagnosis. (PDF)

S5 File Full results of sub-analysis of baseline, comorbidity, and diagnosis model. (PDF)
