# Supplementary material for: Using large administrative data for mining patients’ trajectories for risk stratification: An example from urological diseases
Source: PLoS One. 2024 Nov 13;19(11):e0310981. doi: 10.1371/journal.pone.0310981 (PMC11559980; doi:10.1371/journal.pone.0310981)
Supplement: S1 File — (PDF) [file pone.0310981.s001.pdf]

S1 File. ICD Codes Included as Urological Diseases

N20 - N23 - Stones

N30 - N39 Diseases of the Urinary System

N40 - N53 Diseases of Male Genital Organs

C60 - C69 - Cancers of Urological Diseases

* N390 Urinary tract infection, N393 Urinary Incontinence Female, Male was excluded from analysis.
