# Supplementary material for: Using large administrative data for mining patients’ trajectories for risk stratification: An example from urological diseases
Source: PLoS One. 2024 Nov 13;19(11):e0310981. doi: 10.1371/journal.pone.0310981 (PMC11559980; doi:10.1371/journal.pone.0310981)
Supplement: S2 File — (PDF) [file pone.0310981.s002.pdf]

S2 File. Diagnostic of latent class trajectory model and model selection process.

Using a previously developed framework for latent class trajectory modelling [1,2], the first step was to determine the initial working model based on the residual profile, Bayes information criterion (BIC), and residual standard error. Four models with increasing amounts of polynomials (linear, quadratic, cubic and quartic) were tested (Table 1). It was found that while a quartic polynomial had a lower BIC, the decrease in the standard error of residuals was marginal, hence cubic representation was chosen as the base model.

Table 1: Polynomial Degrees with BIC and Residual Standard Error

Model Degree	BIC	Residual Standard Error
1	313334	0.00224
2	308078	0.00305
3	**305985**	**0.00270**
4	305211	0.00273

* Selected Candidate Model in Bold.

The optimal number of classes (groups) was tested, with testing of one to eight total classes. To ensure that the model chosen was reflective of the data, several model adequacy tests were performed. While the lowest BIC has largely been used as a tool for model selection [3], in some cases, overfitting may cause BIC to further decrease. Hence, other model adequacy methods were also considered [1]. In this study, apart from BIC (Table 2), five main criteria were considered as part of the model adequacy test:

(1) A relative entropy of close to 1 (Table 2).

(2) An entropy of close to 0 (Table 2).

(3) A maximum posterior probability of assignments (APPA) above 70% for all classes (Table 3).

(4) Odds of correct classification above 5.0 for each class (Table 4)

(5) A mismatch of close to zero for each class (Table 5).

Based on the fit criteria and model adequacy tests, 5 different hospitalisation groups were identified, which were assigned based on the posterior probabilities for each group for each patient identified [1].

Table 2: Model Diagnostics including entropy, relative entropy and BIC

Number of Classes	2	3	4	**5***	6	7	8
Entropy	4905.83	34759.97	50676.27	**66842.05**	79975.58	87604.27	102713.11
Relative Entropy	0.93	0.68	0.63	**0.58**	0.55	0.54	0.50
BIC	333583.07	310405.54	298364.17	**289981.32**	285858.86	282793.82	281479.68

* Indicates best model selected.

Table 3: Average posterior probability

Number of Classes	APPA							
2	0.99	0.95	NA	NA	NA	NA	NA	NA
3	0.94	0.71	0.87	NA	NA	NA	NA	NA
4	0.91	0.63	0.78	0.99	NA	NA	NA	NA
**5***	0.69	0.96	0.92	0.56	0.81	NA	NA	NA
6	0.91	0.79	0.74	0.95	0.46	0.48	NA	NA
7	0.44	0.52	0.79	0.85	0.99	0.71	0.73	NA
8	0.66	0.97	0.36	0.84	0.77	0.66	0.71	0.51

* Indicates best model selected.

Table 4: Odds of Correct Classification

Number of Classes	OCC							
2	3.13	412.88	NA	NA	NA	NA	NA	NA
3	821.83	12.07	1.58	NA	NA	NA	NA	NA
4	32.89	35.93	1.46	6719.65	NA	NA	NA	NA
**5***	1.41	4738.3	403.95	13.26	11.3	NA	NA	NA
6	2331.83	31.29	2.59	934.37	1.95	21.68	NA	NA
7	1.78	38.51	63.95	489.85	64800.7	2.67	20.28	NA
8	2.76	2200.3	3.11	1259.72	76.15	23.92	7.33	24.57

* Indicates best model selected.

Table 5: Mismatch

Number of Classes	Mismatch							
2	0.012	-0.012	NA	NA	NA	NA	NA	NA
3	-0.001	-0.096	0.096	NA	NA	NA	NA	NA
4	-0.199	0.01	0.192	-0.003	NA	NA	NA	NA
**5***	**0.264**	**0**	**-0.012**	**-0.03**	**-0.223**	**NA**	**NA**	**NA**
6	0	-0.082	0.026	-0.006	0.059	0.002	NA	NA
7	0.061	0.013	-0.045	0.001	0	0.06	-0.091	NA
8	0.115	-0.003	0.18	0.001	-0.033	-0.058	-0.203	0.001

* Indicates best model selected.

References:

[1] Lennon H, Kelly S, Sperrin M, Buchan I, Cross AJ, Leitzmann M, et al. Framework to construct and interpret latent class trajectory modelling. BMJ Open 2018;8:e020683. https://doi.org/10.1136/bmjopen-2017-020683.

[2] Teuling ND, Pauws S, Heuvel E van den. Clustering of longitudinal data: A tutorial on a variety of approaches 2021.

[3] Nagin DS, Jones BL, Passos VL, Tremblay RE. Group-based multi-trajectory modeling. Stat Methods Med Res 2018;27:2015–23. https://doi.org/10.1177/0962280216673085.
