# Supplementary material for: Using large administrative data for mining patients’ trajectories for risk stratification: An example from urological diseases
Source: PLoS One. 2024 Nov 13;19(11):e0310981. doi: 10.1371/journal.pone.0310981 (PMC11559980; doi:10.1371/journal.pone.0310981)
Supplement: S3 File — (Sub-analysis). (PDF) [file pone.0310981.s003.pdf]

**S3 File. Diagnostic of latent class trajectory model and model selection process for patients with two or more admissions. (Sub-analysis)**

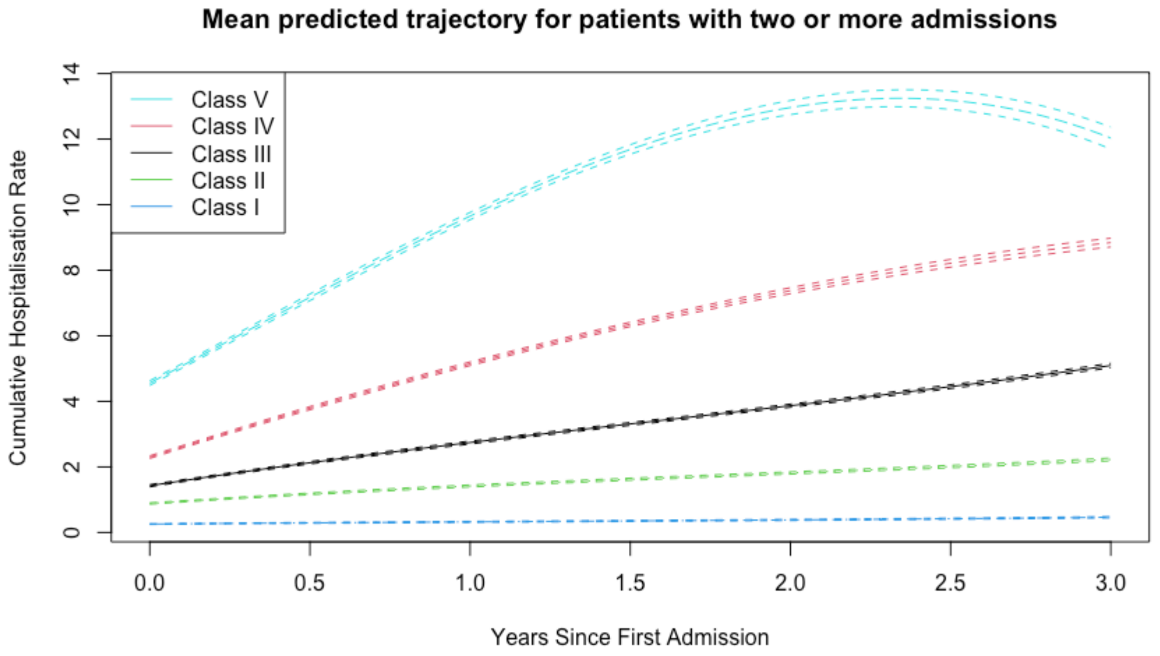


Figure 1 Fitted trajectories for each class for patients with two or more admissions, indicating the trajectory of total hospitalisations that a urology patient may exhibit within 3 years of first hospitalisation, clustered into 5 groups of no (class I), low (class II), medium (class III), medium-high (class IV) and high hospitalisations (class V).

Table 1: Polynomial Degrees with BIC and Residual Standard Error

| Model Degree | BIC | Residual Standard Error |
|---|---|---|
| 1 | 202253.6 | 0.00281 |
| 2 | 199343.0 | 0.00342 |
| 3 | **197896.4** | **0.00321** |
| 4 | 197296.3 | 0.00336 |

* Selected Candidate Model in Bold.

Table 2: Model Diagnostics including entropy, relative entropy and BIC

| Number of Classes | 2 | 3 | 4 | **5*** | 6 | 7 |
|---|---|---|---|---|---|---|
| Entropy | 5046.73 | 12024.79 | 17638.45 | 23061.66 | 27059.15 | 29000.66 |
| Relative Entropy | 0.82 | 0.72 | 0.68 | 0.64 | 0.62 | 0.62 |
| BIC | 217071.09 | 203738.10 | 195973.67 7 | 190299.40 | 187587.45 | 185502.60 |

* Indicates best model selected.

Table 3: Average posterior probability

| Number of Classes | APPA | | | | | | |
|---|---|---|---|---|---|---|---|
| 2 | 0.96 | 0.881 | NA | NA | NA | NA | NA |
| 3 | 0.807 | 0.911 | 0.688 | NA | NA | NA | NA |
| 4 | 0.836 | 0.963 | 0.824 | 0.688 | NA | NA | NA |
| **5*** | 0.534 | 0.888 | 0.754 | 0.747 | 0.91 | NA | NA |
| 6 | 0.852 | 0.874 | 0.801 | 0.845 | 0.558 | 0.479 | NA |
| 7 | 0.564 | 0.984 | 0.827 | 0.729 | 0.388 | 0.696 | 0.751 |

* Indicates best model selected.

Table 4: Odds of Correct Classification

| Number of Classes | OCC | | | | | | |
|---|---|---|---|---|---|---|---|
| 2 | 2.729 | 64.662 | NA | NA | NA | NA | NA |
| 3 | 101.586 | 3.173 | 9.002 | NA | NA | NA | NA |
| 4 | 51.985 | 1467.681 | 2.675 | 6.416 | NA | NA | NA |
| **5*** | 8.939 | 121.369 | 8.106 | 2.527 | 840.656 | NA | NA |
| 6 | 128.668 | 640.002 | 58.886 | 5.783 | 3.276 | 6.757 | NA |
| 7 | 3.321 | 10918.393 | 5.729 | 39.729 | 11.903 | 16.865 | 114.06 |

* Indicates best model selected.

Table 5: Mismatch

| Number of Classes | Mismatch | | | | | | |
|---|---|---|---|---|---|---|---|
| 2 | 0.028 | -0.028 | NA | NA | NA | NA | NA |
| 3 | 0.003 | 0.018 | -0.021 | NA | NA | NA | NA |
| 4 | -0.035 | -0.002 | 0.104 | -0.066 | NA | NA | NA |
| **5*** | 0.012 | -0.024 | -0.134 | 0.145 | 0.001 | NA | NA |
| 6 | -0.012 | 0.001 | -0.038 | -0.02 | 0.052 | 0.015 | NA |
| 7 | 0.047 | 0 | 0.002 | -0.038 | 0.051 | -0.065 | 0.004 |

* Indicates best model selected
