# Supplementary material for: Using large administrative data for mining patients’ trajectories for risk stratification: An example from urological diseases
Source: PLoS One. 2024 Nov 13;19(11):e0310981. doi: 10.1371/journal.pone.0310981 (PMC11559980; doi:10.1371/journal.pone.0310981)
Supplement: S4 File — (PDF) [file pone.0310981.s004.pdf]

S4 File. Full results of baseline, comorbidity and diagnosis model

Table 1: Baseline and comorbidity multinomial logistic regression model for predicting group membership

Variables	Odds Ratio				
	Class I	Class II (95% CI)	Class III (95% CI)	Class IV (95% CI)	Class V (95% CI)
	Baseline Model				
Intercept	1	0.01 (0.0-0.01)*	0.0 (0.0-0.0)*	0.0 (0.0-0.0)*	0.0 (0.0-0.0)*
Age	1	1.02 (1.02-1.02)*	1.03 (1.03-1.03)*	1.02 (1.02-1.02)*	1.0 (1.0-1.0)
CALD	1	1.0 (0.73-1.37)	0.96 (0.7-1.31)	0.94 (0.54-1.63)	0.73 (0.27-1.97)
Charlson score	1	1.05 (1.05-1.05)*	1.07 (1.07-1.07)*	1.12 (1.12-1.12)*	1.14 (1.14-1.14)*
Emergency	1	1.19 (1.1-1.28)*	3.16 (2.98-3.35)*	2.66 (2.42-2.94)*	3.39 (2.78-4.12)*
HACs	1	1.31 (1.19-1.44)*	1.52 (1.41-1.65)*	1.46 (1.27-1.68)*	1.15 (0.89-1.48)
Insurance	1	1.25 (1.17-1.32)*	1.15 (1.08-1.22)*	1.05 (0.95-1.16)	1.15 (0.96-1.37)
Male	1	1.9 (1.75-2.05)*	1.48 (1.37-1.6)*	1.28 (1.12-1.47)*	1.16 (0.92-1.47)
Non-English Pref	1	0.93 (0.68-1.28)	1.17 (0.86-1.61)	1.11 (0.64-1.91)	0.95 (0.34-2.69)
Regional	1	1.04 (0.94-1.15)	0.92 (0.84-1.02)	0.79 (0.66-0.94)*	0.7 (0.5-0.98)*
Rural	1	1.08 (1.02-1.15)*	1.0 (0.94-1.06)	0.94 (0.84-1.06)	1.13 (0.93-1.37)
	Comorbidity Models				
Age	1	1.02 (1.02-1.02)*	1.04 (1.04-1.04)*	1.03 (1.03-1.03)*	1.02 (1.02-1.02)*
CALD	1	1.01 (0.74-1.38)	1.01 (0.75-1.36)	1.02 (0.6-1.73)	0.82 (0.3-2.22)
Cardiovascular disease	1	0.76 (0.6-0.97)*	0.98 (0.84-1.15)	0.73 (0.55-0.98)*	0.87 (0.53-1.42)
Diabetes	1	1.14 (1.05-1.23)*	1.23 (1.16-1.31)*	1.05 (0.93-1.18)	0.95 (0.75-1.2)
Emergency	1	1.35 (1.25-1.46)*	3.63 (3.43-3.85)*	3.82 (3.46-4.21)*	5.47 (4.59-6.53)*
HACs	1	1.58 (1.44-1.75)*	1.9 (1.75-2.05)*	2.32 (2.02-2.66)*	2.05 (1.59-2.65)*
Insurance	1	1.23 (1.16-1.31)*	1.13 (1.06-1.2)*	0.99 (0.9-1.09)	1.03 (0.86-1.23)
Liver disease	1	0.76 (0.31-1.88)	1.09 (0.6-2.01)	1.48 (0.62-3.5)	0.65 (0.09-4.9)
Male	1	1.99 (1.84-2.16)*	1.58 (1.46-1.71)*	1.48 (1.29-1.69)*	1.39 (1.1-1.76)*

**Non-English Preferred**	1	0.96 (0.7-1.31)	1.25 (0.91-1.71)	1.19 (0.68-2.05)	1.04 (0.37-2.94)
**Regional**	1	1.05 (0.95-1.16)	0.93 (0.85-1.03)	0.8 (0.67-0.96)*	0.73 (0.53-1.02)
**Renal disease**	1	1.43 (1.16-1.78)*	1.84 (1.57-2.15)*	2.25 (1.78-2.84)*	2.39 (1.61-3.53)*
**Rural**	1	1.09 (1.03-1.16)*	1.01 (0.95-1.07)	0.96 (0.85-1.08)	1.14 (0.94-1.39)
**Two or more comorbidities**	1	1.02 (0.87-1.19)	1.2 (1.06-1.35)*	0.96 (0.77-1.19)	0.94 (0.65-1.37)

*indicates significant associations (p <0.05)

Table 2 Multiple multinomial logistic regression results and odds ratio for baseline urological diseases without surgical interventions

Disease w/ no surgical interventions	Class I	Class II	Class III	Class IV	Class V
**Benign prostatic hyperplasia**	1	0.84 (0.79-0.89)*	0.87 (0.82-0.92)*	0.39 (0.34-0.45)*	0.24 (0.17-0.33)*
**Bladder cancer**	1	2.69 (2.44-2.97)*	3.0 (2.72-3.31)*	4.57 (3.99-5.24)*	6.17 (4.78-7.96)*
**Bladder-neck obstruction**	1	1.22 (1.04-1.43)*	0.92 (0.77-1.1)	0.45 (0.29-0.7)*	0.17 (0.04-0.67)*
**Calculus of bladder**	1	1.16 (0.99-1.36)	1.38 (1.18-1.61)*	0.9 (0.64-1.25)	0.33 (0.12-0.91)*
**Calculus of kidney**	1	0.93 (0.83-1.05)	0.88 (0.78-0.99)*	0.88 (0.72-1.07)	0.33 (0.19-0.55)*
**Calculus of kidney with calculus of ureter**	1	1.07 (0.86-1.33)	1.02 (0.82-1.27)	0.4 (0.22-0.72)*	0.63 (0.29-1.38)
**Calculus of ureter**	1	0.51 (0.45-0.59)*	0.49 (0.42-0.56)*	0.26 (0.19-0.35)*	0.1 (0.04-0.21)*
**Cystitis**	1	0.58 (0.46-0.74)*	0.53 (0.44-0.65)*	0.34 (0.22-0.52)*	0.21 (0.08-0.54)*
**Disorders of prepuce**	1	0.32 (0.25-0.42)*	0.36 (0.29-0.46)*	0.18 (0.1-0.32)*	0.27 (0.11-0.65)*
**Hydrocele**	1	0.31 (0.22-0.42)*	0.38 (0.29-0.5)*	0.22 (0.12-0.43)*	0.16 (0.04-0.63)*
**Irradiation cystitis**	1	1.4 (1.03-1.92)*	1.36 (1.02-1.83)*	1.95 (1.27-3.01)*	3.16 (1.69-5.91)*
**Kidney cancer**	1	2.1 (1.83-2.4)*	3.82 (3.46-4.21)*	6.3 (5.49-7.22)*	10.38 (8.37-12.88)*
**Other bladder disorders**	1	0.68 (0.61-0.75)*	0.63 (0.57-0.69)*	0.51 (0.41-0.63)*	0.48 (0.32-0.71)*
**Other male genital organ disorders**	1	0.37 (0.28-0.49)*	0.36 (0.28-0.47)*	0.15 (0.07-0.29)*	0.04 (0.0-0.36)*
**Other penis disorders**	1	0.66 (0.44-0.97)*	0.66 (0.46-0.94)*	0.27 (0.1-0.73)*	0.04 (0.0-3.42)
**Other urinary incontinence**	1	0.59 (0.49-0.72)*	0.48 (0.4-0.57)*	0.16 (0.1-0.26)*	0.14 (0.05-0.36)*
**Prostate cancer**	1	1.72 (1.62-1.82)*	1.67 (1.57-1.77)*	3.49 (3.16-3.85)*	3.94 (3.23-4.79)*
**Testicular cancer**	1	0.78 (0.45-1.35)	3.39 (2.38-4.82)*	9.68 (6.41-14.61)*	32.79 (21.3-50.46)*
**Urethral stricture**	1	1.19 (1.07-1.31)*	0.77 (0.67-0.88)*	0.48 (0.36-0.64)*	0.18 (0.07-0.42)*

*indicates significant associations (p <0.05)

Table 3 Multiple multinomial logistic regression results and odds ratio for baseline urological diseases with surgical interventions

Disease w/ surgical interventions	Class I	Class II	Class III	Class IV	Class V
**Benign prostatic hyperplasia**	1	0.48 (0.43-0.54)*	0.32 (0.28-0.37)*	0.15 (0.11-0.19)*	0.12 (0.07-0.19)*
**Bladder cancer**	1	1.52 (1.33-1.75)*	1.11 (0.94-1.29)*	1.65 (1.28-2.13)*	2.77 (1.73-4.44)*
**Bladder-neck obstruction**	1	0.7 (0.58-0.83)*	0.34 (0.27-0.42)*	0.17 (0.1-0.27)*	0.08 (0.02-0.33)*
**Calculus of bladder**	1	0.66 (0.56-0.79)*	0.51 (0.42-0.62)*	0.33 (0.22-0.48)*	0.15 (0.05-0.45)*
**Calculus of kidney**	1	0.53 (0.45-0.62)*	0.32 (0.28-0.38)*	0.32 (0.24-0.42)*	0.15 (0.08-0.29)*
**Calculus of kidney with calculus of ureter**	1	0.61 (0.48-0.78)*	0.38 (0.29-0.48)*	0.15 (0.08-0.27)*	0.29 (0.12-0.7)*
**Calculus of ureter**	1	0.29 (0.24-0.34)*	0.18 (0.15-0.21)*	0.09 (0.06-0.13)*	0.04 (0.02-0.1)*
**Cystitis**	1	0.34 (0.26-0.43)*	0.2 (0.16-0.25)*	0.13 (0.08-0.21)*	0.09 (0.03-0.27)*
**Disorders of prepuce**	1	0.18 (0.14-0.24)*	0.13 (0.1-0.17)*	0.06 (0.03-0.12)*	0.12 (0.05-0.32)*
**Hydrocele**	1	0.17 (0.12-0.24)*	0.14 (0.1-0.19)*	0.08 (0.04-0.16)*	0.07 (0.02-0.31)*
**Irradiation cystitis**	1	0.8 (0.58-1.12)*	0.5 (0.37-0.69)*	0.71 (0.44-1.16)*	1.46 (0.71-3.02)*
**Kidney cancer**	1	1.2 (1.0-1.43)*	1.42 (1.21-1.66)*	2.34 (1.81-3.02)*	4.9 (3.12-7.7)*
**Other bladder disorders**	1	0.41 (0.36-0.47)*	0.25 (0.21-0.29)*	0.2 (0.15-0.28)*	0.24 (0.14-0.42)*
**Other male genital organ disorders**	1	0.21 (0.16-0.28)*	0.13 (0.1-0.17)*	0.05 (0.03-0.11)*	0.02 (0.0-0.17)*
**Other penis disorders**	1	0.38 (0.25-0.57)*	0.24 (0.17-0.35)*	0.1 (0.04-0.27)*	0.02 (0.0-1.58)*
**Other urinary incontinence**	1	0.34 (0.27-0.42)*	0.18 (0.14-0.22)*	0.06 (0.03-0.1)*	0.06 (0.02-0.17)*
**Prostate cancer**	1	1.09 (0.97-1.23)*	0.68 (0.59-0.78)*	1.75 (1.38-2.21)*	2.51 (1.63-3.86)*
**Testicular cancer**	1	0.44 (0.26-0.77)*	1.26 (0.87-1.83)*	3.6 (2.25-5.76)*	15.8 (8.78-28.45)*
**Urethral stricture**	1	0.67 (0.58-0.77)*	0.29 (0.24-0.35)*	0.18 (0.13-0.27)*	0.09 (0.03-0.22)*

*indicates significant associations ($p < 0.05$)
