# Supplementary material for: Using large administrative data for mining patients’ trajectories for risk stratification: An example from urological diseases
Source: PLoS One. 2024 Nov 13;19(11):e0310981. doi: 10.1371/journal.pone.0310981 (PMC11559980; doi:10.1371/journal.pone.0310981)
Supplement: S5 File — (PDF) [file pone.0310981.s005.pdf]

S5 File. Full results of sub-analysis of baseline, comorbidity, and diagnosis model

<u>Sub-analysis of patients with two or more admissions</u>

Similar trajectories were shown for patients with two or more admissions when compared to the trajectories for all patients (Figure 1).

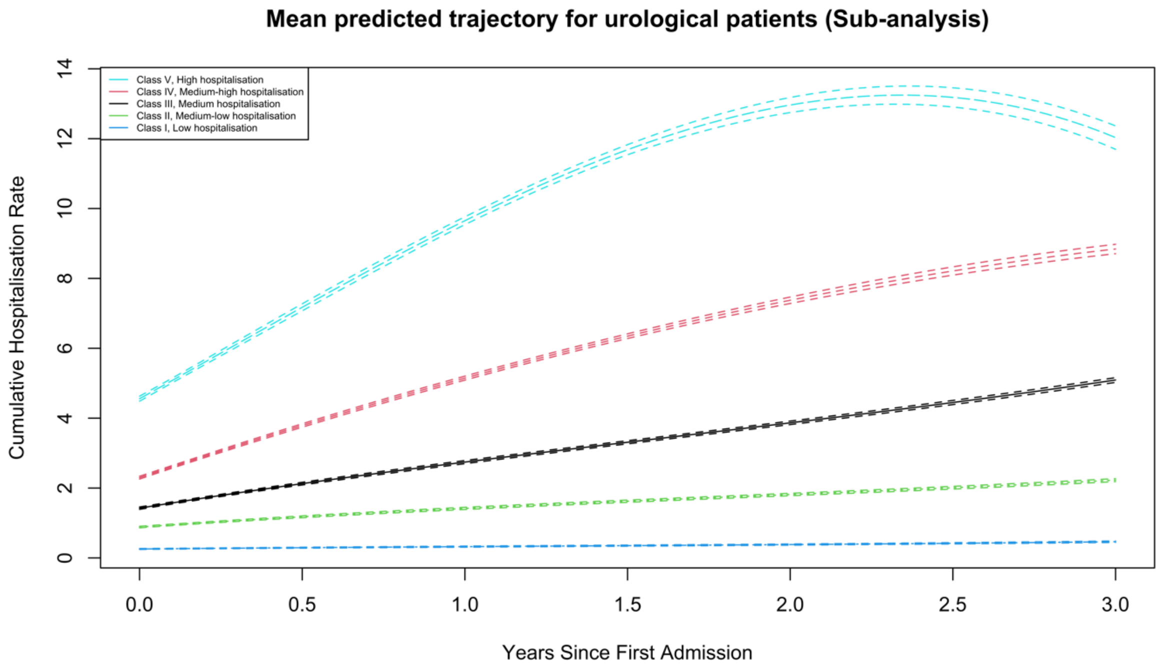

Figure 1: Sub-analysis trajectories of patients diagnosed with urological diseases

Table 1: Baseline and comorbidity multinomial logistic regression model for predicting group membership (sub-analysis).

Variables	Odds Ratio				
	Class I	Class II (95% CI)	Class III (95% CI)	Class IV (95% CI)	Class V (95% CI)
	**Baseline Model**				
**Intercept**	1	0.0 (0.0-0.0)*	0.01 (0.01-0.01)*	0.0 (0.0-0.0)*	0.0 (0.0-0.0)*
**Age**	1	1.05 (1.05-1.05)*	1.03 (1.03-1.03)*	1.04 (1.04-1.04)*	1.02 (1.02-1.02)*
**CALD**	1	1.07 (0.75-1.53)	1.02 (0.73-1.42)	0.99 (0.54-1.82)	0.78 (0.28-2.16)
**Charlson score**	1	1.12 (1.12-1.12)*	1.06 (1.06-1.06)*	1.17 (1.17-1.17)*	1.21 (1.19-1.23)*
**Emergency**	1	6.23 (5.76-6.74)*	2.25 (2.08-2.43)*	5.47 (4.87-6.16)*	6.96 (5.83-8.3)*
**HACs**	1	4.76 (4.23-5.35)*	3.74 (3.33-4.21)*	4.44 (3.72-5.29)*	3.74 (2.85-4.93)*
**Insurance**	1	1.05 (0.99-1.11)	1.12 (1.05-1.18)*	0.99 (0.88-1.11)	1.08 (0.91-1.29)
**Male**	1	0.98 (0.89-1.08)	1.32 (1.2-1.46)*	0.79 (0.68-0.93)*	0.76 (0.59-0.98)*
**Non-English Pref**	1	1.34 (0.92-1.94)	1.08 (0.78-1.51)	1.23 (0.66-2.31)	1.08 (0.38-3.12)
**Regional**	1	0.91 (0.81-1.03)	1.03 (0.93-1.14)	0.8 (0.66-0.98)*	0.71 (0.51-0.99)*
**Rural**	1	1.03 (0.95-1.11)	1.04 (0.96-1.13)	0.96 (0.84-1.1)	1.11 (0.89-1.37)
	**Comorbidity Models**				
**Age**	1	1.05 (1.05-1.05)*	1.04 (1.04-1.04)*	1.05 (1.05-1.05)*	1.04 (1.04-1.04)*
**CALD**	1	1.09 (0.77-1.56)	1.0 (0.72-1.4)	1.08 (0.6-1.95)	0.96 (0.36-2.56)
**Cardiovascular disease**	1	3.25 (2.47-4.28)*	2.72 (2.03-3.65)*	2.36 (1.63-3.43)*	3.03 (1.79-5.15)*
**Diabetes**	1	1.26 (1.16-1.36)*	1.21 (1.12-1.31)*	1.09 (0.95-1.26)	0.98 (0.77-1.24)
**Emergency**	1	5.21 (4.81-5.63)*	1.92 (1.77-2.07)*	5.16 (4.58-5.8)*	6.89 (5.78-8.22)*
**HACs**	1	4.9 (4.36-5.52)*	3.63 (3.23-4.09)*	5.47 (4.59-6.53)*	5.1 (3.96-6.59)*
**Insurance**	1	1.03 (0.97-1.09)	1.12 (1.05-1.18)*	0.91 (0.81-1.03)	0.96 (0.81-1.15)
**Liver disease**	1	19.11 (4.39-83.1)*	13.07 (2.89-59.1)*	34.12 (7.11-163.69)*	11.36 (0.94-136.89)
**Male**	1	1.15 (1.04-1.27)*	1.45 (1.31-1.6)*	1.02 (0.87-1.19)	1.04 (0.82-1.32)
**Non-English Pref**	1	1.43 (0.99-2.08)	1.11 (0.79-1.54)	1.39 (0.76-2.55)	1.34 (0.48-3.7)

**Regional**	1	0.95 (0.85-1.07)	1.05 (0.95-1.16)	0.84 (0.69-1.03)	0.76 (0.54-1.05)
**Renal disease**	1	5.42 (4.2-6.99)*	3.9 (2.96-5.13)*	6.11 (4.47-8.36)*	6.96 (4.52-10.71)*
**Rural**	1	1.08 (1.0-1.17)	1.07 (0.99-1.16)	1.03 (0.9-1.18)	1.2 (0.97-1.49)
**Two or more comorbidities**	1	3.29 (2.76-3.92)*	2.61 (2.19-3.12)*	2.41 (1.87-3.11)*	2.53 (1.71-3.75)*

* indicates significant associations (p <0.05)

Table 2 Sub-analysis multiple multinomial logistic regression results and odds ratio for baseline urological diseases with non surgical interventions

Disease w/ no surgical interventions	Class I	Class II	Class III	Class IV
**Benign prostatic hyperplasia**	1	1.55 (1.44-1.68)*	1.72 (1.59-1.86)*	0.73 (0.62-0.85)*
**Bladder cancer**	1	1.6 (1.42-1.8)*	1.35 (1.22-1.49)*	2.39 (2.04-2.79)*
**Bladder-neck obstruction**	1	1.13 (0.91-1.4)	1.42 (1.21-1.66)*	0.51 (0.31-0.84)*
**Calculus of bladder**	1	1.36 (1.12-1.66)*	1.28 (1.1-1.5)*	0.9 (0.62-1.31)
**Calculus of kidney**	1	0.59 (0.52-0.68)*	0.6 (0.53-0.68)*	0.51 (0.4-0.64)*
**Calculus of kidney with calculus of ureter**	1	0.52 (0.41-0.65)*	0.53 (0.42-0.67)*	0.2 (0.11-0.37)*
**Calculus of ureter**	1	0.23 (0.2-0.27)*	0.28 (0.24-0.32)*	0.1 (0.07-0.15)*
**Cystitis**	1	0.93 (0.72-1.2)	0.96 (0.76-1.22)	0.51 (0.31-0.85)*
**Disorders of prepuce**	1	0.64 (0.48-0.86)*	0.73 (0.55-0.96)*	0.35 (0.19-0.65)*
**Hydrocele**	1	0.65 (0.45-0.94)*	0.86 (0.63-1.18)	0.55 (0.28-1.07)
**Irradiation cystitis**	1	1.14 (0.8-1.62)	1.12 (0.8-1.56)	1.65 (1.03-2.64)*
**Kidney cancer**	1	7.69 (6.7-8.82)*	3.97 (3.4-4.65)*	12.68 (10.63-15.13)*
**Other bladder disorders**	1	0.68 (0.61-0.77)*	0.84 (0.76-0.93)*	0.58 (0.46-0.74)*
**Other male genital organ disorders**	1	1.55 (1.11-2.17)*	1.63 (1.19-2.23)*	0.5 (0.21-1.18)
**Other penis disorders**	1	1.6 (0.98-2.61)	1.46 (0.91-2.34)	0.54 (0.17-1.76)
**Other urinary incontinence**	1	1.49 (1.18-1.89)*	1.7 (1.37-2.11)*	0.52 (0.3-0.88)*
**Prostate cancer**	1	0.95 (0.88-1.03)	0.84 (0.78-0.91)*	2.01 (1.79-2.27)*
**Testicular cancer**	1	4.85 (3.28-7.19)*	0.93 (0.56-1.55)	13.33 (8.49-20.92)*
**Urethral stricture**	1	0.61 (0.52-0.72)*	0.95 (0.85-1.07)	0.33 (0.23-0.46)*

*indicates significant associations ($p < 0.05$)

Table 3 Sub-analysis multiple multinomial logistic regression results and odds ratio for baseline urological diseases with surgical interventions

Disease w/ surgical interventions	Class I	Class II	Class III	Class IV
**Benign prostatic hyperplasia**	1	0.43 (0.37-0.5)*	0.7 (0.62-0.78)*	0.23 (0.17-0.3)*
**Bladder cancer**	1	0.47 (0.39-0.56)*	0.58 (0.51-0.67)*	0.74 (0.56-0.97)*
**Bladder-neck obstruction**	1	0.33 (0.25-0.42)*	0.61 (0.51-0.72)*	0.16 (0.09-0.27)*
**Calculus of bladder**	1	0.4 (0.31-0.5)*	0.55 (0.46-0.66)*	0.28 (0.18-0.43)*
**Calculus of kidney**	1	0.17 (0.14-0.21)*	0.25 (0.22-0.3)*	0.15 (0.11-0.21)*
**Calculus of kidney with calculus of ureter**	1	0.15 (0.11-0.2)*	0.23 (0.18-0.3)*	0.06 (0.03-0.12)*
**Calculus of ureter**	1	0.07 (0.05-0.08)*	0.12 (0.1-0.14)*	0.03 (0.02-0.05)*
**Cystitis**	1	0.27 (0.2-0.37)*	0.41 (0.32-0.54)*	0.16 (0.09-0.27)*
**Disorders of prepuce**	1	0.19 (0.13-0.26)*	0.31 (0.23-0.42)*	0.11 (0.05-0.21)*
**Hydrocele**	1	0.19 (0.13-0.28)*	0.37 (0.27-0.52)*	0.17 (0.08-0.34)*
**Irradiation cystitis**	1	0.33 (0.23-0.48)*	0.48 (0.34-0.69)*	0.51 (0.3-0.84)*
**Kidney cancer**	1	2.1 (1.72-2.55)*	1.67 (1.4-1.99)*	3.53 (2.63-4.73)*
**Other bladder disorders**	1	0.21 (0.17-0.25)*	0.37 (0.32-0.43)*	0.19 (0.14-0.26)*
**Other male genital organ disorders**	1	0.45 (0.32-0.65)*	0.71 (0.51-0.99)*	0.15 (0.06-0.37)*
**Other penis disorders**	1	0.47 (0.28-0.78)*	0.63 (0.39-1.03)*	0.17 (0.05-0.55)*
**Other urinary incontinence**	1	0.43 (0.33-0.57)*	0.73 (0.58-0.93)*	0.16 (0.09-0.28)*
**Prostate cancer**	1	0.27 (0.23-0.32)*	0.35 (0.31-0.39)*	0.76 (0.59-0.98)*
**Testicular cancer**	1	1.42 (0.94-2.14)*	0.4 (0.24-0.67)*	4.14 (2.53-6.75)*
**Urethral stricture**	1	0.18 (0.15-0.23)*	0.41 (0.35-0.49)*	0.11 (0.07-0.16)*

*indicates significant associations (p <0.05)
